# What frog gill resorption brings: loss of function, cell death, and metabolic reorganization

**DOI:** 10.1186/s12983-024-00532-4

**Published:** 2024-04-16

**Authors:** Liming Chang, Wei Zhu, Jianping Jiang

**Affiliations:** 1 Chinese Academy of Sciences Key Laboratory of Mountain Ecological Restoration and Bioresource Utilization & Ecological Restoration and Biodiversity Conservation Key Laboratory of Sichuan Province, Chengdu Institute of Biology, Chinese Academy of Sciences, Chengdu, 610041 China; 2 University of Chinese Academy of Sciences, Beijing, 100049 China

**Keywords:** Gill resorption, Gill respiration, Metabolic switch, Amphibian, Metamorphosis

## Abstract

**Background:**

Anuran metamorphosis, which is driven by thyroid hormone (TH)-mediated processes, orchestrates intricate morphological and functional transformations for the transition from aquatic tadpoles to terrestrial life, providing a valuable model for studying organ functionalization, remodeling, and regression. Larva-specific organ regression is one of the most striking phenomena observed during the anuran metamorphic climax. While previous studies extensively analyzed the regression mechanisms of the tail, the molecular processes governing gill resorption remain elusive.

**Results:**

We employed *Microhyla fissipes* as a model, and utilized a comprehensive approach involving histological analysis, transmission electron microscopy, and transcriptomics to unravel gill development and resorption. The pro-metamorphic stages revealed highly developed gill structures, emphasizing their crucial role as the primary respiratory organ for tadpoles. The transcriptomic analysis highlighted the upregulation of genes associated with enhanced respiratory efficiency, such as hemoglobin and mucins. However, as metamorphosis progressed, gill filaments underwent shrinkage, decreases in blood vessel density, and structural changes that signified a decline in respiratory function. The molecular mechanisms driving gill resorption involved the TH pathway—in particular, the upregulation of thyroid hormone receptor (TR) β, genes associated with the tumor necrosis factor pathway and matrix metalloproteinases. Two distinct pathways orchestrate gill resorption, involving apoptosis directly induced by TH and cell death through the degradation of the extracellular matrix. In addition, metabolic reorganization during metamorphosis is a complex process, with tadpoles adapting their feeding behavior and mobilizing energy storage organs. The gills, which were previously overlooked, have been unveiled as potential energy storage organs that undergo metabolic reorganization. The transcriptomic analysis revealed dynamic changes in metabolism-related genes, indicating decreased protein synthesis and energy production and enhanced substrate transport and metabolism during metamorphic climax.

**Conclusion:**

This study sheds light on the structural, molecular, and metabolic dynamics during gill development and resorption in *M. fissipes*. The findings deepen our understanding of the intricate mechanisms governing organ regression and underscore the pivotal role of the gills in facilitating the transition from aquatic to terrestrial habitats.

## Background

Anuran metamorphosis, which is a thyroid hormone (TH)-mediated process, instigates rapid and profound morphological and functional transformations in multiple organ systems to prepare aquatic tadpoles for terrestrial life [1]. For example, the locomotor organ transitions from the tail to the limbs, to adapt to terrestrial motion; the respiratory system shifts from gills to lungs to address the challenges of the breathing medium’s transition from water to air; the digestive system undergoes restructuring to accommodate changes in diet, and the neural system is remodeled to enhance responsiveness in the intricate terrestrial environment [2–5]. This orchestrated metamorphosis showcases a comprehensive array of changes across various organ systems, providing a valuable model for studying organ functionalization, remodeling, and regression.

Larva-specific organ (i.e., tail and gill) regression is one of the most striking phenomena observed during the anuran metamorphic climax. At present, the processes and molecular mechanisms of tail regression have been comprehensively analyzed and characterized [6, 7]. Wang et al. (2019) revealed the gene expression program underlying tail resorption during the metamorphosis of *Microhyla fissipes* [8]; Yoshio (2019) systematically elucidated the molecular processes of tail resorption in *Xenopus* tadpoles [9]; Wang et al. (2022) illustrated the morphology and molecular mechanisms of tail resorption during metamorphosis in *Rana chensinensis* tadpoles [10]. Overall, tail regression in anurans is orchestrated through two distinct mechanisms: "suicide" and "murder". In the "suicide" pathway, tissues such as muscles, the spinal cord, blood vessels, the outer notochord sheath, and the epidermis undergo apoptosis that is directly induced by TH. Meanwhile, in the "murder" pathway, cell death result from the degradation of the extracellular matrix and the loss of cellular anchorage [11, 12]. These pathways collectively lead to the collapse of the notochord, contraction of the surviving slow muscles, and, ultimately, the orchestrated loss of the tail. In addition, previous studies have demonstrated that TH can autonomously induce the degeneration of the gills [13]. During this process, the gills exhibit a reduction in weight and a decrease in vascularity [14, 15]. However, the molecular mechanisms governing gill resorption remain largely elusive. The unveiling of the molecular mechanisms associated with gill absorption and their comparison with the mechanisms involved in tail absorption are pivotal for deepening our comprehension of the processes underlying organ regression. This exploration significantly contributes to a more nuanced understanding of the intricate mechanisms that regulate cell death.

Metabolic reorganization is a complex process that occurs during anuran metamorphosis. During the pre- and pro-metamorphic stages, tadpoles exhibit voracious feeding behaviors to acquire substantial resources for growth and energy storage [16]. During the metamorphic climax, tadpoles curtail their food intake to facilitate the remodeling of the gastrointestinal tract [17]. To meet the extensive biosynthetic and energy demands associated with organogenesis and organ remodeling, tadpoles have to mobilize the metabolic reorganization of energy storage organs (i.e., the fat body and liver) [18, 19]. Zhu et al. (2020) revealed that the tail can also be an energy storage organ during the metamorphic climax. The metabolic flux from the apoptotic tail can replace hepatic fat storage as a metabolic fuel, resulting in increased hepatic amino acid and fat levels [20]. Tadpole gills also undergo apoptosis during the metamorphic climax. However, whether gills can serve as an energy storage organ while undergoing metabolic reorganization to support tadpole metamorphosis remains poorly understood.

*Microhyla fissipes* (Anura: Microhylidae) is a suitable model for exploring the processes of adaption from aquatic to terrestrial environments during metamorphosis given its distinct aquatic and terrestrial life stages, abundant population, rapid developmental rate, and clear genetic background. In this study, we employed an integrated approach involving histological sectioning, transmission electron microscopy (TEM), and transcriptomics to investigate gill development and resorption during the pro-metamorphic stages (stages 37–41) and metamorphic climax (stage 43). Our comprehensive analysis elucidates the structural and functional alterations in the gills, unveiling the molecular mechanisms and metabolic reorganization underlying gill resorption. This research underscores the pivotal role of the gills in facilitating the amphibian transition from aquatic to terrestrial habitats during metamorphosis.

## Materials and methods

### Animal and daily culture

*Microhyla fissipes* adults were collected from farmlands (E103.459885°, N30.744614°, 701 m) located in Shifang City, Sichuan Province, China. Adult male and female frogs were bred according to the standard procedures for artificially induced spawning and egg production [21]. Four egg clutches, which ranged from 200 to 500 eggs each, were collected and placed in twelve aquatic containers (length 42 × width 30 × depth 10 cm, water depth = 5 cm). The eggs were allowed to hatch at a temperature of 25 ± 0.5 °C with a light/dark cycle of 12:12 hours. Upon hatching, the larvae were initially fed a solution of boiled chicken egg yolk once per day for two days. Subsequently, tadpoles were fed spirulina powder (from China National Salt Industry Corporation) once a day, and the water in the containers was replaced every two days. The developmental stages of the tadpoles were determined using the staging table reported by Wang et al. (2017) [21]. To a facilitate comprehensive comparison of developmental stages across various amphibian species, we established correspondences between different amphibian developmental tables (Table 1).

**Table 1:** The correspondences between different amphibian developmental tables

**Developmental phase**	**Anuran stage number (Gosner, 1960) **[22]	***Xenoups* stage number (Nieuwkoop and Faber, 1994) **[23]	***M. fissipes* stage number (Wang et al., 2017) **[21]	**Developmental features**
Pro-metamorphosis	37	55	37	All toes completely separated
Pro-metamorphosis	38	56-57	38-39	Metatarsal tubercle
Pro-metamorphosis	39	57	40	Subarticular tubercles
Pro-metamorphosis	40	58	40	Disappearance of vent tube I
Pro-metamorphosis	41	59	41	Disappearance of vent tube II
Metamorphic climax	42	60-61	42	Emergence of forelimbs
Metamorphic climax	43	62	42-43	Tail degeneration I
Metamorphic climax	44	63	43	Tail degeneration II
Metamorphic climax	45	64-65	44	Tail degeneration III
Post-metamorphosis	46	66	45	Complete metamorphosis

### Experimental design and sampling

**Fig. 1:**
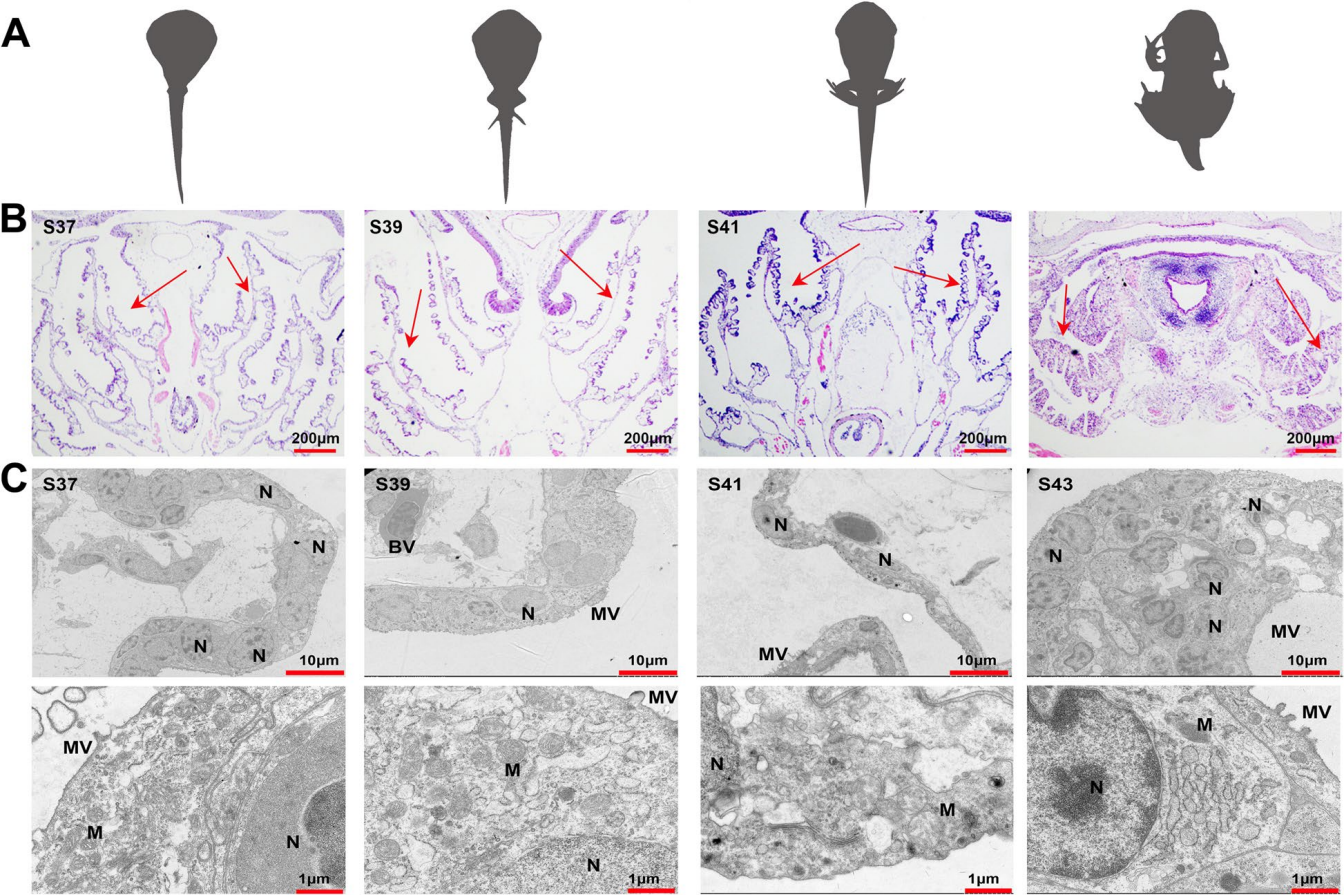
Structural characteristics of the gills in different developmental stages. **A** Schematic diagram showing the sampling stages of this study. **B** Histological characteristics of the gills (*n* = 3 each stage), the red arrows indicating the gill filaments. **C** Ultrastructural characteristics of the gills, *BV* blood vessel, *M* mitochondria, *Mv* microvillus, *N* cell nucleus

*Microhyla fissipes* individuals were collected from stage 37 (29 days postfertilization, pf), stage 39 (34 days pf), stage 41 (39 days pf), and stage 43 (42 days pf) (Fig. 1A). After individuals were euthanized with Tricaine (MS-222), the gill tissues were collected for histological sections, transmission electron microscopy (TEM), and RNA-seq experiments. Each experiment involved four developmental stages and three replicates of each developmental stage to ensure their robustness and reliability. In the histological sections and TEM experiments, each replicate consisted of a single individual gill. To ensure the attainment of the minimum amount of tissue required for RNA extraction, a strategic sample merging approach was implemented. At developmental stages 37, 39, 41, and 43, each replicate consisted of 30, 20, 15, and 10 individual gills, respectively.

### Histological analysis

The method of histological sectioning followed the protocol described by Chang et al. (2021) [24]. Histological sections were stained via hematoxylin–eosin staining (HE). Section staining followed the instructions of commercial kits purchased from Servicebio Technology Co., Ltd. (Wuhan, China). After sealing with resinene, the histological sections were photographed using a Nikon E200 microscope equipped with an industrial digital camera (APTINA CMOS Sensor, San Jose, USA).

### Transmission electron microscopic (TEM) observation

Gill tissues were collected and diced into small blocks of 1 mm^3^ each. These tissue blocks underwent fixation in 3% glutaraldehyde for 6 hours at 4 °C. Post-fixation involved rinsing the tissue blocks three times in 0.1 M Sorensen’s phosphate buffer (pH 7.4) for 15 minutes each, followed by a 2-hour post-fixation in 1% osmium tetroxide within the same buffer. Additional rinsing and dehydration were carried out using a graded ethanol series (30, 50, 70, 80, 95, and 100%, each for 20 minutes). Following overnight penetration with a mixture of acetone and EMBed 812 at 37 °C, the tissue blocks were embedded in EMBed 812. The resin and sample-containing embedding models were polymerized in an oven at 65 °C for over 48 hours. The resulting resin blocks were finely sectioned into ultrathin slices ranging from 60 to 80 nm using an ultramicrotome (Leica UC7) and Diamond slicer (Daitome Ultra 45°). These ultrathin sections were placed onto 150-mesh cuprum grids with a formvar film and stained for 8 minutes in a 2% uranium acetate saturated alcohol solution. Following rinsing with 70% ethanol and ultrapure water, the ultrathin sections underwent an additional 8-minute staining in a 2.6% lead citrate solution. After drying with filter paper, the cuprum grids were arranged on a grid board and left to dry overnight at room temperature. Finally, the cuprum grids were observed under a transmission electron microscope (TEM) (Hitachi, HT7800/HT7700) operating at 60 kV, and images were captured using a digital CCD camera.

### Transcriptomic analyses

Gill samples were promptly fresh-frozen in liquid nitrogen and stored at -80 °C until RNA extraction. The procedure for total RNA extraction followed the established protocol for TRIzol (Life Technologies Corp., Carlsbad, CA, USA). Subsequently, 1 μg of RNA from each sample was utilized for library construction by employing the NEBNext®Ultra™ RNA Library Prep Kit for Illumina® (NEB, USA). Sequencing was performed on an Illumina Hiseq 2000 platform from Biomarker Technologies Co. Ltd. to generate paired-end reads. The raw sequencing data were deposited in the Genome Sequence Archive (GSA) under the accession number PRJCA004230. Clean data were obtained by filtering out reads containing adapters, poly-N, and low-quality reads from the raw dataset. De novo assembly of transcriptome was accomplished using Trinity [25], and subsequent annotation was conducted by querying against various databases, including NR (NCBI non-redundant protein sequences), Pfam (protein family), KOG/COG/eggNOG (clusters of orthologous groups of proteins), Swiss-Prot (a manually annotated and reviewed protein sequence database), KEGG (Kyoto Encyclopedia of Genes and Genomes), and GO (Gene Ontology). Gene expression levels were quantified using RSEM [26], and analyses of gene differential expression between developmental stages were conducted using DESeq2 [27]. Significantly differentially expressed genes (DEGs) were identified based on a stringent criterion of q < 0.05 after Benjamini and Hochberg’s correction. Then, gene enrichment analysis was conducted based on the KEGG database using KOBAS 3.0 with an E-value threshold of 1.0E-5 [28].

### Weighted correlation network analysis (WGCNA)

In this study, we employed a WGCNA to elucidate gene clusters or modules associated with specific developmental stages [29]. The method involved constructing a scale-free network using gene expression profiles. Initially, a similarity matrix was established by calculating the absolute value of Pearson’s correlation coefficient between gene pairs based on gene expression data. This similarity matrix was then transformed into an adjacency matrix, and a soft threshold (β value) was incorporated to accentuate strong connections and diminish weak correlations. The next step involved converting the adjacency matrix into a topological matrix (TOM) to quantify the strength of associations between genes. The TOM served as input for a hierarchical clustering analysis, and the dynamic tree-cut algorithm was applied to identify network modules. Module eigengenes (MEs) that represented the overall gene expression level within the modules were identified as the first principal components. Module membership (MM) was assessed through Pearson’s correlation coefficient between a gene expression profile across all samples and the ME. Simultaneously, the gene significance (GS) value was utilized to evaluate genes with developmental stage information, where a higher GS value indicated greater representativeness for a specific developmental stage. Core genes that met stringent criteria (GS > 0.7 and MM > 0.7), were selected to unveil the biological functions of these modules. This rigorous gene selection process aimed to provide a more in-depth understanding of the biological implications associated with each developmental stage, forming a robust foundation for our research findings.

## Results

### Structural changes in the gills during metamorphosis

The gills of *M. fissipes* exhibited notable structural changes across the developmental stages. The histological analysis revealed that in stage 37, the gill filaments exhibited a highly branched structurethat was covered with capillaries. The branching of the gill filaments gave rise to numerous projections on both sides, which further developed into secondary projections. As the development progressed to stage 41, the gill filaments began to aggregate. Finally, in stage 43, the gill filaments fully coalesced and contracted into a compact mass (Fig. 1B). The results of transmission electron microscopy (TEM) indicated that in stages 37 and 39, the gill epithelial cells exhibited an elliptical shape with round nuclei and abundant cytoplasmic mitochondria. Microvilli were evident on the cell surface, along with surface-active substances that were detected on the outer side, while blood vessels were distributed along the inner side of the epithelial layer. As the development progressed to stage 41, a reduction in mitochondrial abundance was observed, and it was accompanied by a noticeable shrinkage and flattening of the gill epithelial cells. The blood vessels could still be observed. By stage 43, a striking transformation occurred, and it was characterized by irregularly shaped, shrunken epithelial cells. The nuclei became condensed and smaller. Additionally, some cells exhibited vacuoles within the cytoplasm, and blood vessels were not observed. These findings offer valuable insights into the dynamic cellular changes during gill development (Fig. 1C).

### Functional analysis of co-expressed modules in the gills during metamorphosis

**Fig. 2:**
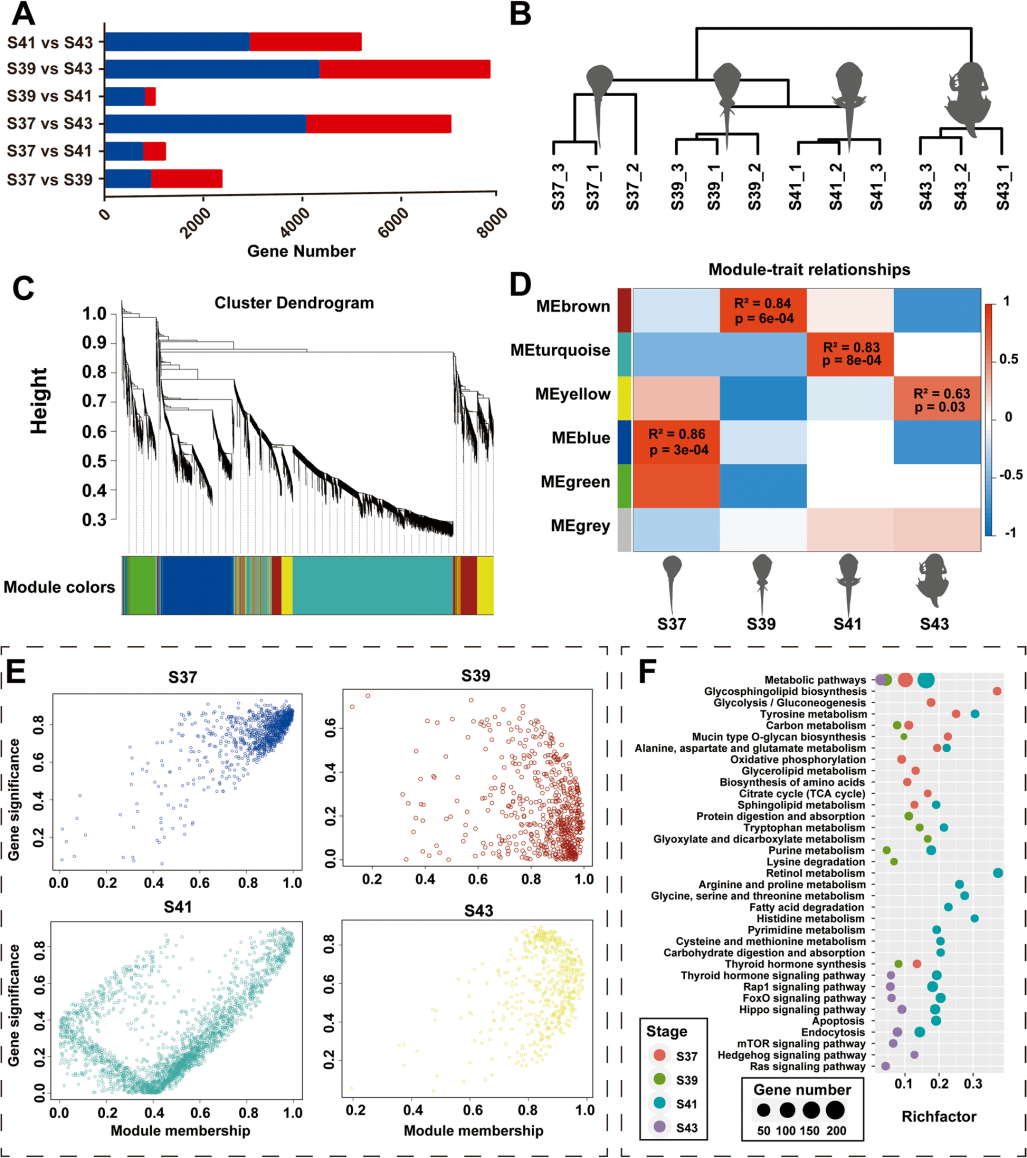
Functional enrichment of gene modules in gills during metamorphosis. **A** The DEGs identified in all pairwise comparisons of stages; (**B**) Hierarchical clustering results of gill samples in different stages; (**C**) Hierarchical clustering results of DEGs; (**D**) The correlations between module eigengenes and developmental phases. The color scale indicates the strength of correlation; (**E**) The featured genes of gills in different developmental stages; (**F**) The main functional items of enriched gene modules (FDR < 0.05) in pro-metamorphosis (S37, S39, and S41) and metamorphosis climax (S43) (*N*=3 each stage)

In this study, the numbers of DEGs in pairwise comparisons between the four stages—namely, S37 vs. S39, S37 vs. S41, S37 vs. S43, S39 vs. S41, S39 vs. S43, and S41 vs. S43—were 2,387, 1,246, 7,014, 1,040, 7,803, and 5,202, respectively (Fig. 2A). The hierarchical clustering analysis based on the DEGs revealed that the samples from each developmental stage were clustered together, with samples from stages 37 and 39 being clustered first, followed by those from stage 41, while the samples from stage 43 formed a distinct cluster (Fig. 2B). Furthermore, a co-expression network analysis based on the DEGs identified four gene co-expression modules (Fig. 2C and D). The blue, brown, turquoise, and yellow modules were significantly positively correlated with the pro-metamorphic S37, S39, and S41 and the metamorphic S43, respectively (*p-value* < 0.05 and R^2^ > 0.6, Fig. 2E). The functional enrichment analysis of the core gene sets in these modules revealed that the genes featured in the pro-metamorphosis stage (S37–39) were mainly enriched in metabolic pathway (Fig. 2F). In contrast, the featured genes in the metamorphic climax (S44) were mainly enriched in signaling pathways that regulate cell activities, such as the Rap1, FoxO, Hippo, mTOR, Hedgehog, Ras signaling pathways, as well as endocytosis pathways, that collectively regulated cell proliferation, differentiation, and apoptosis (Fig. 2F). Furthermore, the genes featured in S41 showed enrichments in both metabolic pathways and signaling pathways regulating cell activities. In addition, the TH synthesis and TH signaling pathways were highlighted in S37–39 and S41–43, respectively.

### The transcriptional patterns of respiratory function-related proteins

**Fig. 3:**
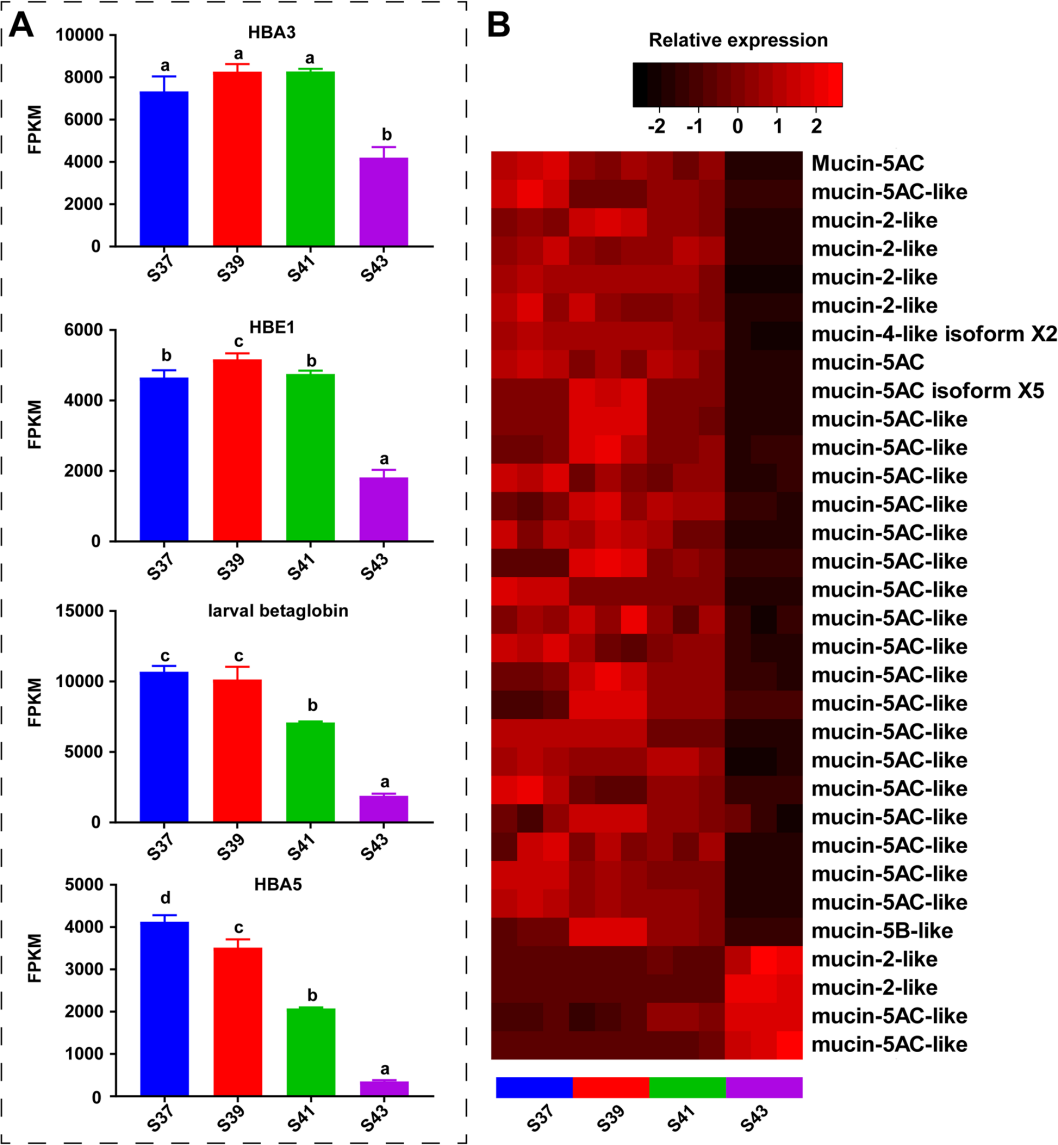
The transcriptional switches of respiratory function related proteins. **A**-**B** The transcriptional patterns of HBs (**A**) and mucin (**B**). Different letters denote significance between groups at a threshold of *p value* < 0.05 (one-way ANOVA and S-N-K post-hoc test)

In this study, we present a comprehensive analysis of the expression patterns of hemoglobin and mucin genes during metamorphosis. Four distinct hemoglobin transcripts were identified, namely, *HBE1*, *HBA5*, *HBA3*, and larval β globin, and they had temporal expression patterns that varied significantly throughout metamorphosis. Notably, *HBE1* and *HBA3* exhibited high transcriptional levels during the pro-metamorphic stages (S37–41), followed by a sharp decrease at the metamorphic climax (S43). HBA5 and larval β globin displayed a gradual decrease in expression from S37 to S43 (Fig. 3A). Additionally, we identified 32 mucin genes that could be classified into five major categories: mucin-5AC, mucin-5B, mucin-2, mucin-4, and mucin-19. Strikingly, the expression of four mucin genes was highly specific at the metamorphic climax (S43), while 28 mucin genes showed specifically high expression during the pro-metamorphic stages (S39–41) (Fig. 3B).

### The molecular processes of cell death during gill resorption

**Fig. 4:**
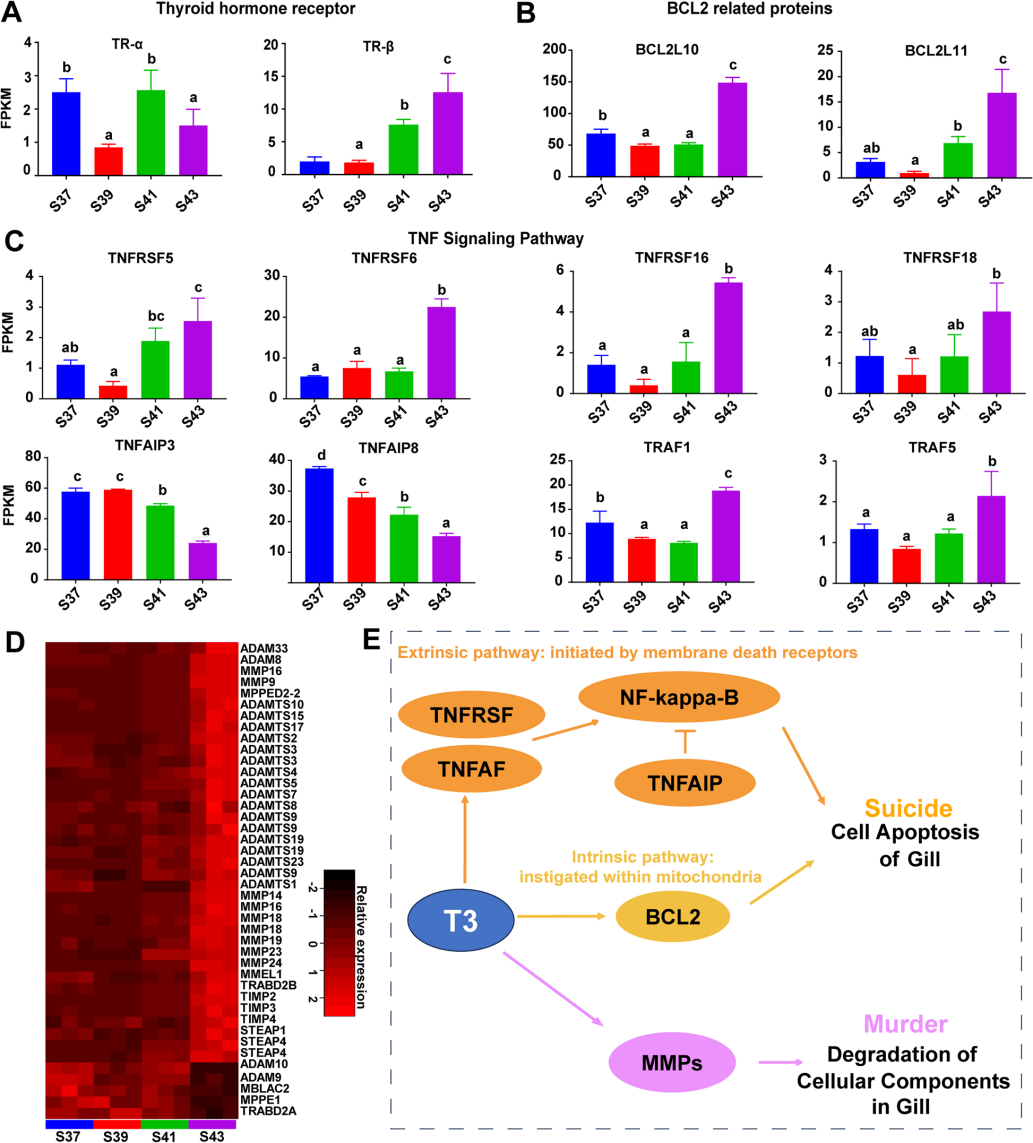
The transcriptional patterns of gill apoptosis related genes. **A**-**C** bar plots showing the DEGs involving in thyroid hormone receptors (TRs) (**A**), BCL-2 related proteins (**B**), and the tumor necrosis factor (TNF) signaling pathway (**C**). Different letters denote significance between groups at a threshold of *p value* < 0.05 (one-way ANOVA and S-N-K post-hoc test). **D** Heat maps presenting the variation patterns of genes that are involved in matrix metalloproteinase (gene expression varied with developmental stages, *p value* < 0.05, one-way ANOVA). **E** Schematic diagram of the pathway involved in gill resorption

The DEGs associated with gill apoptosis were analyzed to investigate the molecular mechanisms underlying gill resorption during metamorphosis. This study identified two thyroid hormone receptor (TR) genes; of these, *TRβ* demonstrated an increasing transcriptional trend throughout development, peaking at S43, while *TRα* exhibited relatively low transcriptional levels from S37 to S41 (Fig. 4A). Additionally, two BCL2-related genes (*BCL2L10* and *BCL2L11*) and six genes related to the tumor necrosis factor (TNF) signaling pathway (*TNFRSF5*, *TNFRSF6*, *TNFRSF16*, *TNFRSF18*, *TRAF1*, and *TRAF5*) displayed similar transcriptional patterns. These genes maintained low transcriptional levels during pro-metamorphosis (S37–S41) and exhibited an increase in their transcriptional levels at the metamorphic climax (S43). In contrast, two genes related to the TNF signaling pathway (*TNFAIP3* and *TNFAIP8*) displayed high transcriptional levels during pro-metamorphosis and a subsequent decrease at the metamorphic climax (Fig. 4B and C).

### The metabolic switches during gill resorption

**Fig. 5:**
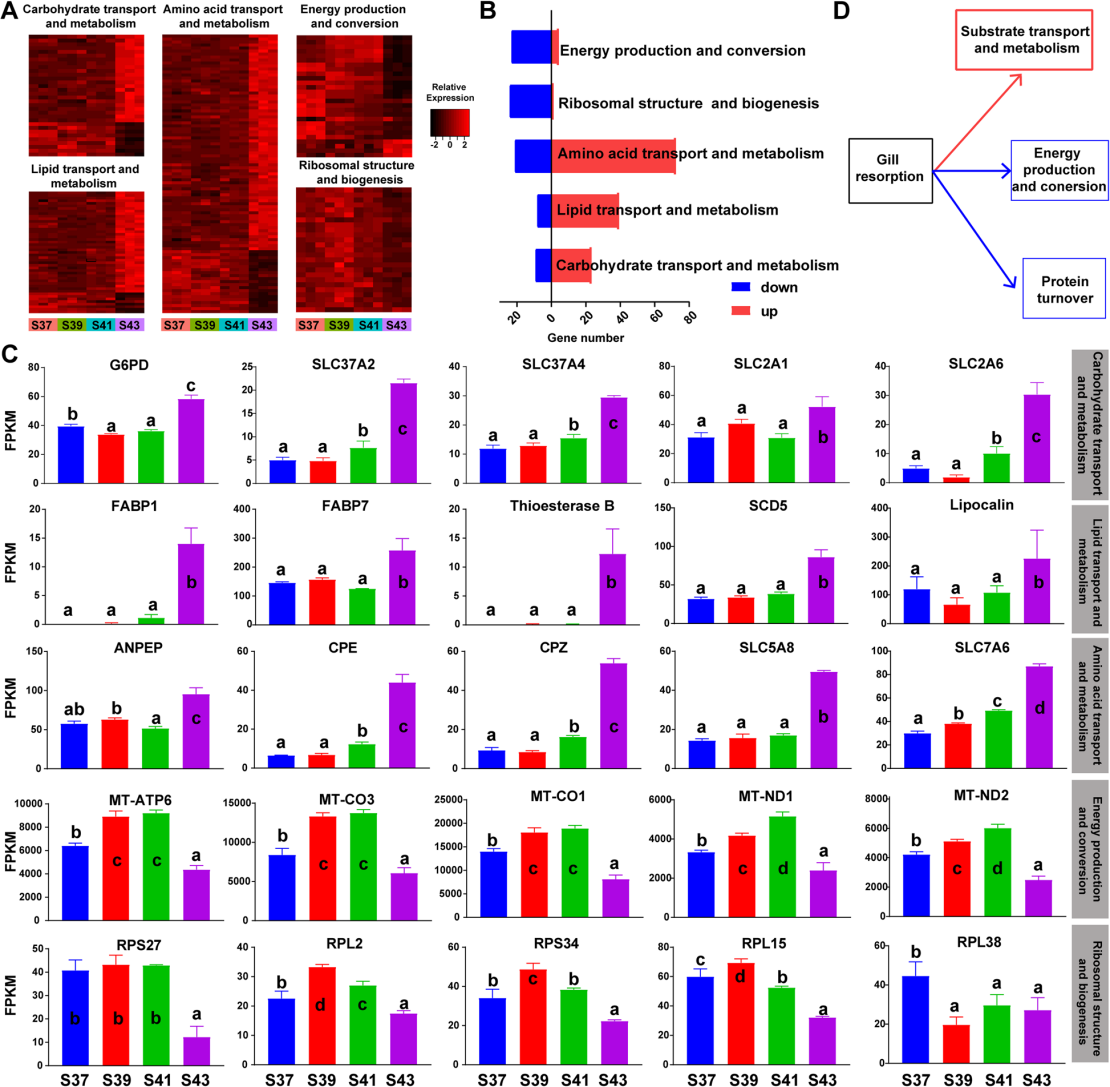
The transcriptional switches of metabolic genes during gill apoptosis. **A** Heat maps presenting the variation patterns of metabolic genes (gene expression varied with developmental stages, *p* < 0.05, one-way ANOVA). **B** Statistics of the numbers of up-regulated and down-regulated metabolic genes at metamorphic climax. **C** The transcriptional patterns of metabolic genes. Different letters denote significance between groups at a threshold of *p value* < 0.05 (one-way ANOVA and S-N-K post-hoc test). **D** Schematic map explaining the metabolic switches during gill apoptosis. Arrows in red and blue colors denote upregulation and downregulation, respectively

The transcriptional patterns of metabolic genes across various developmental stages showed noteworthy alterations that coincided with the onset of the metamorphic climax. Specifically, this developmental stage exhibited significant transcriptional changes in various metabolic gene categories: 47 lipid transport and metabolism genes (downregulated:upregulated = 8:39), 32 carbohydrate transport and metabolism genes (downregulated:upregulated = 9:23), 93 amino acid transport and metabolism genes (downregulated:upregulated = 21:72), 25 ribosomal protein genes (downregulate:upregulated = 24:1), and 27 genes associated with energy production and conversion (downregulated:upregulated = 23:4) (Fig. 5A and B). Moreover, the core genes related to substrate transport and metabolism were prominently featured in S44. Conversely, the core genes associated with energy production and conversion, as well as those involved in ribosomal structure and biogenesis, exhibited the lowest transcriptional levels in S44 (Fig. 5C).

## Discussion

In this study, we employed an integrated approach by combining histological analyses encompassing both conventional histological sections and TEM with transcriptomics to study the structural changes in and molecular processes of gill resorption during metamorphosis. Our results showed that gill resorption in *M. fissipes* is intricately linked with a cascade of physiological changes, including the loss of respiratory functionality, cell death, and a restructuring of metabolic processes in the gills during the metamorphic climax. These findings underscore the synchronized orchestration of events that are pivotal to the successful progression of metamorphosis in frogs.

### The change from gill to lung respiration during metamorphosis

In pro-metamorphic stages, frog tadpoles rely on their gills as the primary respiratory organ, and they undergo significant degeneration, disappearing nearly entirely [14] or leaving only a small remnant [30] by the time the metamorphic climax is reached. In this study, we observed extensive structural differentiation in the gills of *M. fissipes* from S37 to 39. This was characterized by highly branched gill filaments that were densely covered with capillaries. The projections of the gill filaments featured a surface layer of pseudostratified columnar epithelial cells, which significantly augmented the contact area with gases (Fig. 1B). Ultrastructural studies of gill epithelial cells during this stage revealed the presence of microvilli, substantial substance secretion, and numerous mitochondria in the cytoplasm, which was indicative of robust respiratory function.

By stage 41, we observed the onset of gill filament shrinkage, reduced spacing between filaments, and contraction of gill epithelial cells (Fig. 1C). Remarkably, despite these changes, the overall gill structure remained intact, suggesting effective gas exchange in water during this stage. As metamorphosis progressed, the gill filaments continued to shrink and the blood vessel density decreased, particularly at the metamorphic climax. Gill epithelial cells assumed irregular shapes, which was accompanied by condensation and the reduction in size of cell nuclei. Additionally, a decrease in mitochondrial presence and the appearance of cytoplasmic cavities were noted (Fig. 1C). Consistently with these observations, the gills in *R. catesbeiana* at Gosner stage 44 (metamorphic climax) showed decreased weight and reduced vascularization [14]. Microvascular casting in *Xenopus laevis* gills further revealed that the branching of gill filament row veins reached its maximum at Nieuwkoop and Faber (NF) stage 58, and they gradually decreased in number and frequency until NF stage 62 (metamorphic climax) [15]. This suggested an accelerated regression of the blood–air barrier in the gills during the metamorphic climax. Meanwhile, significant morphological changes occurred in the lungs of *M. fissipes*, including an increase in alveolar septa, leading to the division of the lung parenchyma into numerous irregular compartments. Additionally, there was a noticeable proliferation in the number and elongation of cilia on alveolar epithelial cells, which was accompanied by an augmented presence of capillaries traversing the alveolar wall. Furthermore, a reduction in the intercellular distances between alveolar epithelial and endothelial cells was evident, which was indicative of the establishment of a mature blood-gas barrier in the lungs [5].

The transcriptomic analysis of the gills unveiled dynamic gene expression patterns, which notably highlighted the significant upregulation of hemoglobin and mucin genes during pro-metamorphosis, contrasting with their lowest expression levels observed during the metamorphic climax (Fig. 3A and B). Hemoglobin, a protein that is predominantly found in red blood cells, plays a pivotal role in the transport of oxygen and carbon dioxide [31, 32]. The heightened expression of hemoglobin genes during pro-metamorphosis implies an augmented capacity for gas exchange, contributing to the respiratory efficiency of the gills during this developmental stage. Mucins, which are characterized by their high molecular weight and extensive glycosylation, are synthesized by epithelial tissues in most animals. Serving as integral components of numerous gel-like secretions, mucins fulfill diverse functions, including lubrication, acting as a protective barrier against pathogens and toxic substances, and maintaining the hydration layer of the epithelium. Moreover, their role as a permeable gel layer facilitates the exchange of gases and nutrients between the upper epithelium and the underlying layers [33, 34]. The substantial upregulation of mucin genes in tadpole gills during pro-metamorphosis underscores their active participation in respiratory processes, further supporting the notion of a robust respiratory function. Conversely, the observed decline in the expression levels of hemoglobin and mucin genes during metamorphic climax suggests a diminishing respiratory capacity of the gills. This reduction aligns with the structural changes in gill filaments. Simultaneously, transcriptional switches of functional proteins for respiration in the lungs (i.e., pulmonary surfactant proteins and hemoglobin) occur during the metamorphic climax, which is indicative of an augmentation of respiratory function [5]. In summary, our findings elucidate the temporal continuity between the loss of gill respiratory function and the enhancement of lung respiratory capacity during frog development, underscoring the dynamic interplay between respiratory organs during frog metamorphosis.

### The molecular mechanisms underlying cell death in gill resorption

TH is the key factor in initiating cell death pathways during amphibian metamorphosis [7]. It was evidenced that TH exerted its effects via the TH receptor (TR), and there was a pair of TR subtypes encoded by separate genes, *TRα* and *TRβ*. Our results showed that as metamorphosis proceeded, the transcriptional level of *TRβ* in the gills of *M. fissipes* increased and peaked in metamorphic climax (Fig. 4A). It has been shown that the treatment of pre-metamorphic tadpoles with a *TRβ*-specific agonist, GC-1, induced gill resorption and tail shortening in vivo [35]. These findings suggest that the apoptosis of larval cells in the gills is predominantly mediated by *TRβ*. In addition, we observed the upregulation of the transcription of BCL2-related genes (*BCL2*-like protein 10 and *BCL2*-like protein 11) in S44 (Fig. 4B). The *BCL2* protein family plays a crucial role in determining cellular apoptosis, which is vital for organ development, tissue homeostasis, and immune function [36, 37]. The upregulation of transcription levels of *BCL2*-related genes suggested the activation of the process of apoptosis during the metamorphic climax.

Furthermore, the TNF pathway plays a significant role in inducing programmed cell death, and the TNF receptor superfamily has been proven to be the major death receptor involved in the extrinsic pathway of tail apoptosis in *X. laevis* tadpoles [38, 39]. Based on the transcriptomic analysis, we identified four major categories of genes associated with the TNF signaling pathway: members of the TNF receptor superfamily (*TNFRSF5*, *TNFRSF6B*, *TNFRSF16*, *TNFRSF18*, and *TNFRSF19*), TNF alpha-induced proteins (*TNFAIP2*, *TNFAIP3*, and *TNFAIP8*), and TNF receptor-associated factors (*TRAF1* and *TRAF5*) (Fig. 4C). Among these, the binding of *TNFSF5/C40LG* to *TNFRSF5* can activate extracellularly regulated protein kinases in macrophages and B cells to induce immunoglobulin secretion [40]. *TNFRSF16* can mediate neuronal cell death [41]. *TNFRSF18* is involved in the interaction between activated T lymphocytes and endothelial cells, and it regulates T-cell receptor-mediated cell death and activates the NF-kappa-B pathway through the TRAF2/NIK pathway [42]. By mediating the activation of the JNK and NF-kappa-B pathways, *TNFRSF19* further promotes caspase-independent cell death [43]. Both *TRAF1* and *TRAF5* participate in regulating the activation of the NF-kappa-B and JNK pathways, playing a role in the process of regulating cell apoptosis [44, 45]. The upregulation of these genes suggests the activation of the TNF signaling pathway during the metamorphic climax. *TNFAIP3* can interact with TRAF1/TRAF2 and inhibit the activation of the NF-kappa-B pathway [46, 47]. Meanwhile, the transcription of *TNFAIP3* and *TNFAIP8* in the gills of *M. fissipes* tadpoles was downregulated in S44. Considering that *TNFAIP8* can inhibit the activity of caspase-8, thereby negatively regulating TNF-mediated apoptosis [48], these results further support the activation of the TNF signaling pathway and apoptosis during the metamorphic climax.

Matrix metalloproteinases (MMPs) are a type of collagenase and belong to the metzincin superfamily. They act as extracellular-matrix-degrading enzymes that are responsible for breaking down various protein components of the extracellular matrix during tissue apoptosis [49]. The upregulation of matrix metalloproteinases during the metamorphic climax similarly suggested their involvement in the absorption processes in the gills during this stage (Fig. 4D).

In summary, we illuminated the intricate processes involved in gill resorption through two distinct mechanisms that are analogous to the phenomenon of tail regression and are denoted as "suicide" and "murder" [11, 12] (Fig. 4E). In the former, apoptosis is triggered directly by the action of TH in gill tissues. Within this context, our study delineated two primary apoptotic pathways: the extrinsic pathway, which is initiated by membrane death receptors, such as the TNF receptor superfamily, and the intrinsic pathway, which is instigated within mitochondria by members of the BCL2 family. This dual-pathway model underscores the complexity of the molecular cascades governing cellular apoptosis in the context of gill regression. Furthermore, we also delineated a distinct facet of gill resorption characterized as "murder", wherein cell death ensues through the degradation of the extracellular matrix and the consequential loss of cellular anchorage. These findings enhanced our understanding of the regulatory dynamics of gill resorption, offering insights into the intricate interplay of the molecular pathways and cellular events that govern this biological process.

### The metabolic switches during gill resorption

The gills undergo metabolic changes during both the execution of respiratory functions and the process of significant resorption, and these involve alterations in both substance and energy metabolism. Based on the results of the transcriptomic analysis, we explored the transcriptional patterns of metabolism-related genes in the gills at different developmental stages. Ribosomal protein genes, including *RPS27*, *PPS34*, *RPL2*, *RPL15*, and *RPL38*, which are crucial constituents of ribosomes, exhibited transcriptional upregulation during the pre-metamorphic stages (S37 to 39) but underwent downregulation at the metamorphic climax (S43) (Fig. 5C). This upregulation suggests the augmentation of cellular protein synthesis capabilities, which is indicative of concurrent gill tissue growth alongside tadpole development during the pro-metamorphic stages. Respiration is a process entailing energy production and storage. The core genes that are integral to the mitochondrial respiratory chain, including ATP synthase subunit (*MT-ATP6*), NADH-ubiquinone oxidoreductase chains (*MT-ND1* and *MT-ND2*), and cytochrome c oxidase subunits (*MT-CO1* and *MT-CO3*), maintained elevated expression levels in the gills. This sustained upregulation is possibly consistent with the increased respiratory demands in tadpoles during the pro-metamorphic stages (Fig. 5C). In contrast to the energy metabolism, most genes related to substrate metabolism maintained relatively low transcription levels during the pro-metamorphic stages and experienced substantial upregulation during the metamorphic climax. For instance, G6PD catalyzed the rate-limiting step of the oxidative pentose–phosphate pathway, offering an alternative route for carbohydrate dissimilation beyond glycolysis [50]. Glucose-6-phosphate exchanger (*SLC37A2* and *SLC37A4*) may transport cytoplasmic glucose-6-phosphate into the lumen of the endoplasmic reticulum [51], while facilitated glucose transporter members of the solute carrier family 2 (*SLC2A1* and *SLC2A6*) exhibited the ability to transport a wide range of aldoses, including both pentoses and hexoses [52]. The transcriptional upregulation of these genes pointed to enhanced carbohydrate transport and metabolism. Additionally, genes involved in lipid transport and metabolism, including fatty-acid-binding proteins (*FABP1* and *FABP7*), *SCD5*, thioesterase B, and lipocalin [53, 54], as well as those associated with amino acid transport and metabolism, such as aminopeptidase (*ANPEP*), carboxypeptidase (*CPE* and *CPZ*), sodium-coupled monocarboxylate transporter (*SLC5A8*), and Y+L amino acid transporter (*SLC7A6*) [55–58], exhibited similar expression patterns (Fig. 5C). Enhanced substrate transport and metabolism align with the rapid apoptosis observed in the gills during the metamorphic climax, wherein many macromolecules undergo hydrolysis, producing corresponding substrate molecules. The enhanced metabolism and transport of substrates facilitate the translocation of substrate molecules to other rapidly growing and metabolically active organs during tissue apoptosis (Fig. 5D). Therefore, during the metamorphic climax, the gills, which serve as an energy-supplying organ, recycle substrate molecules through the resorption process, providing metabolic fuel for the frog's metamorphic development.

## Data Availability

The sequencing data in this study have been submitted to the Genome Sequence Archive (GSA; https://bigd.big.ac.cn/gsa) under accession number PRJCA004230.
